# Improving Dengue Forecasts by Using Geospatial Big Data Analysis in Google Earth Engine and the Historical Dengue Information-Aided Long Short Term Memory Modeling

**DOI:** 10.3390/biology11020169

**Published:** 2022-01-21

**Authors:** Zhichao Li, Helen Gurgel, Lei Xu, Linsheng Yang, Jinwei Dong

**Affiliations:** 1 Key Laboratory of Land Surface Pattern and Simulation, Institute of Geographic Sciences and Natural Resources Research, Chinese Academy of Sciences, Beijing 100101, China; lizc@igsnrr.ac.cn (Z.L.); yangls@igsnrr.ac.cn (L.Y.); 2 Department of Geography, University of Brasilia (UnB), Brasilia 70910-900, Brazil; helengurgel@unb.br; 3 Vanke School of Public Health, Tsinghua University, Beijing 100084, China; xu_lei@mail.tsinghua.edu.cn

**Keywords:** dengue, Google Earth Engine, LSTM, geospatial big data, risk forecasting

## Abstract

**Simple Summary:**

Forecasting dengue cases often face challenges from (1) time-effectiveness due to time-consuming satellite data downloading and processing, (2) weak spatial representation due to data dependence on administrative unit-based statistics or weather station-based observations, and (3) stagnant accuracy without historical dengue cases. With the advance of the geospatial big data cloud computing in Google Earth Engine and deep learning, this study proposed an efficient framework of dengue prediction at an epidemiological week basis using geospatial big data analysis in Google Earth Engine and Long Short Term Memory modeling. We focused on the dengue epidemics in the Federal District of Brazil during 2007–2019. Based on Google Earth Engine and epidemiological calendar, we computed the weekly composite for each dengue driving factor, and spatially aggregated the pixel values into dengue transmission areas to generate the time series of driving factors. A multi-step-ahead Long Short Term Memory modeling was used, and the time-differenced natural log-transformed dengue cases and the time series of driving factors were considered as outcomes and explantary factors, respectively, with two modeling scenarios (with and without historical cases). The performance is better when historical cases were used, and the 5-weeks-ahead forecast has the best performance.

**Abstract:**

Timely and accurate forecasts of dengue cases are of great importance for guiding disease prevention strategies, but still face challenges from (1) time-effectiveness due to time-consuming satellite data downloading and processing, (2) weak spatial representation capability due to data dependence on administrative unit-based statistics or weather station-based observations, and (3) stagnant accuracy without the application of historical case information. Geospatial big data, cloud computing platforms (e.g., Google Earth Engine, GEE), and emerging deep learning algorithms (e.g., long short term memory, LSTM) provide new opportunities for advancing these efforts. Here, we focused on the dengue epidemics in the urban agglomeration of the Federal District of Brazil (FDB) during 2007–2019. A new framework was proposed using geospatial big data analysis in the Google Earth Engine (GEE) platform and long short term memory (LSTM) modeling for dengue case forecasts over an epidemiological week basis. We first defined a buffer zone around an impervious area as the main area of dengue transmission by considering the impervious area as a human-dominated area and used the maximum distance of the flight range of *Aedes aegypti* and *Aedes albopictus* as a buffer distance. Those zones were used as units for further attribution analyses of dengue epidemics by aggregating the pixel values into the zones. The near weekly composite of potential driving factors was generated in GEE using the epidemiological weeks during 2007–2019, from the relevant geospatial data with daily or sub-daily temporal resolution. A multi-step-ahead LSTM model was used, and the time-differenced natural log-transformed dengue cases were used as outcomes. Two modeling scenarios (with and without historical dengue cases) were set to examine the potential of historical information on dengue forecasts. The results indicate that the performance was better when historical dengue cases were used and the 5-weeks-ahead forecast had the best performance, and the peak of a large outbreak in 2019 was accurately forecasted. The proposed framework in this study suggests the potential of the GEE platform, the LSTM algorithm, as well as historical information for dengue risk forecasting, which can easily be extensively applied to other regions or globally for timely and practical dengue forecasts.

## 1. Introduction

Dengue fever is a mosquito-borne viral disease mainly transmitted in urban and suburban areas in tropical and subtropical regions worldwide and tends to expand to new areas [1,2]. A dengue early warning system (EWS) permits the accurate forecasting of dengue outbreaks in advance and provides sufficient time to implement preventive measures [3], which often requires routine access to dengue data collected within administrative units [4,5] and a set of climate and environmental factors affecting the number and spatial distribution of dengue mosquito vectors (i.e., *Aedes aegypti* and *Aedes albopictus*), such as rainfall, air temperature, relative humidity data from in situ observations, and normalized difference vegetation index (NDVI) from remote sensing [6,7,8]. However, efficient and accurate dengue forecasting faces challenges. First of all, data downloading and processing takes a large amount of time, which hinders the time-effective generation of time series of various climate and environmental factors. Second, the spatial representation and matching of cases, and driver data, are different. Dengue cases were often collected from administrative unit-based statistics, while the climate data are dependent on meteorological observations and vegetation data are from spatially explicit remote sensing data.

Thanks to the rapid development of remote sensing and cloud computing techniques, dengue-related climate and environmental factors can be collected and processed based on geospatial big data as well as via the cloud-based platform of Google Earth Engine (GEE) [9,10,11]. The GEE platform integrates multi-sensor satellite images, ready-to-use datasets, and various algorithms (e.g., image preprocessing, image composite-visual interpretation, feature extraction, traditional machine learning, and deep learning) [12,13,14]. The GEE has been used to identify the driving factors of malaria transmission and is proven to be useful to generate climate and environmental factors and match with spatio-temporal resolutions of epidemiological data [15,16]; however, it has not been used in dengue risk forecasting yet. 

Numerous factors contribute to the spread of dengue through human populations that causes non-stationarity in dengue cases time series (i.e., the features of the dynamical epidemiological processes evolve with time) [17,18]. Despite this, historical dengue information is one of the useful features for forecasting future dengue risk [5]. In terms of the models of dengue case forecasting, autoregressive integrated moving average (ARIMA), machine learning (ML), and deep learning (DL), have been widely used in previous studies and ARIMA is often used as a benchmark to evaluate the performance of other models [4,5,6,7,8,19,20,21]. The ARIMA is a univariate linear model that needs stationary input time series. Using ARIMA, the stationarity of dengue data time series should be fully investigated by multiple statistical tests (e.g., the Augmented Dickey–Fuller (ADF) test and the Kwiatkowski–Phillips–Schmidt–Shin (KPSS) test [22]), and the non-stationarity should be removed to obtain a better forecast performance in real applications. Recently, long short term memory (LSTM) has become the most active and effective network for forecasting the dengue risk and shows good performance [5,6,23] as it can use multivariate time series as input features, and learn the nonlinearities and long-term dependencies in time series [24]. Although LSTM is not sensitive to the non-stationarity of time series, making features and target time series stationary will reduce the prediction complexity and improve forecast accuracy, especially in real applications with limited length of time series of dengue cases.

The Federal District of Brazil (FDB) was selected as the study area, which was created in 1960 to house the new national capital, Brasilia, with rapid urbanization and population growth in the past decades [25]. The urban agglomeration in the FDB has become the third-largest metropolis in Brazil, and has been greatly affected by dengue epidemics in past years [26,27]. In Brazil, the Notifiable Diseases Information System (SINAN) is the official portal for entering and processing reported dengue cases. According to the dengue cases in the FDB collected by SINAN, the dengue epidemic presents a seasonal pattern, and the annual incidence steadily increases [27]. A great dengue outbreak was observed in 2019, with 47,745 reported cases [27]. However, to our knowledge, the model of dengue risk prediction has not been established to date. Moreover, weather stations are insufficient and the spatial distribution is uneven in the FDB [28], which also hinders the implementation of accurate dengue risk prediction.

In this context, taking the FDB as study area, this study aims to propose a novel framework of dengue risk forecasting based on cloud-based analyses of geospatial big data in the GEE platform and historical information-aided LSTM modeling. Specifically, this study expects to make three important contributions: (1) time series of climate and environmental factors were processed using GEE-based analysis of geospatial big data. It showcased the potential of cloud computing and geospatial big data for timely dengue forecasting to a broader audience in public health; (2) historical dengue cases were considered in LSTM modeling; (3) a forecast of dengue cases at an epidemiological week (namely epi week) basis was proposed, focusing on the epidemics during 2007–2019 and considering the epidemic during 2018–2019 as the outcome. 

## 2. Materials and Methods

**Figure 1:**
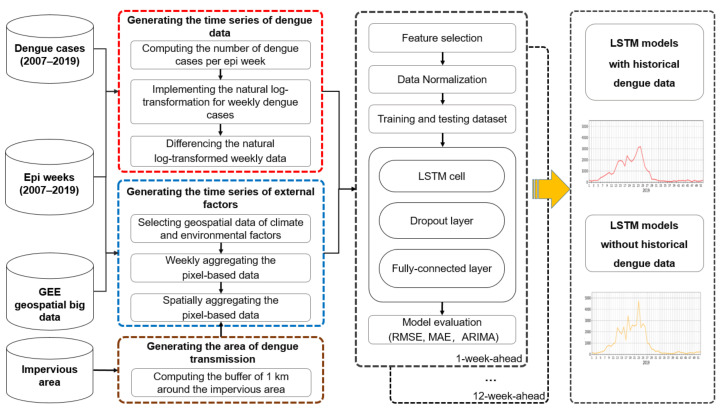
The framework of dengue risk forecasting based on the analysis of geospatial big data in GEE and LSTM modeling.

A new framework of weekly dengue case forecasting using GEE and LSTM was proposed (Figure 1). It includes (i) defining the epi weeks during the study period (i.e., 2007–2019) and generating a stationary time series of weekly dengue data; (ii) defining the main area of dengue transmission and computing the time series of climate and environmental factors based on the analysis of geospatial big data in the GEE platform; (iii) implementing 1-week to 12-week ahead forecasts that consider different time lags (i.e., 1 to 12 weeks) in advance of dengue epidemics and evaluating LSTM models. The detailed information is presented as follows.

### 2.1. Study Area and Dengue Cases

**Figure 2:**
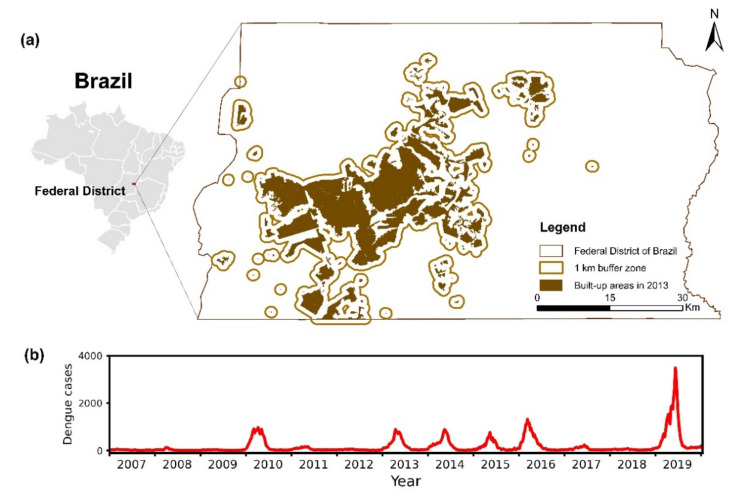
Geolocation of the Federal District of Brazil (**a**) and the number of dengue cases per epidemiological week during 2007–2019 (**b**). The impervious land indicates the human-dominated area, and the buffer zone of 1 km indicates the main area of dengue transmission.

This study was carried out in the FDB, with fragmented and unevenly distributed impervious land (Figure 2a). The significant urban expansion and population growth in the past decades make dengue an important public health issue. In this study, dengue cases from 2007 to 2019 were obtained from the Notifiable Diseases Information System (SINAN) database, the official portal for entering and processing reported dengue cases throughout Brazil [26,27,29]. In this region, suspected cases from healthcare units (i.e., public hospitals, emergency units, basic healthcare units, private hospitals, and private laboratory) were confirmed by the central laboratories and the health regions. We computed the dengue case count per epi week for the FDB, and a time series of 678 weekly dengue case count values during 2007–2019 was generated (Figure 2b). We computed the natural log-transformation for weekly dengue cases plus one and then the difference between two consecutive time steps (Equation (1)) to obtain a stationary time series of weekly dengue data as a dependent factor (namely time-differenced log-transformed weekly dengue cases). In the processing of epidemiological data, both the ADF and KPSS tests were used to examine the stationarity:

(1) $$D_{t}=\log(N_{t}+1)-\log(N_{t-1}+1)$$

where *D_t_* represents the time-differenced natural log-transformed weekly dengue cases, *N_t_* and *N_t-_*_1_ represent the dengue cases per epi week at time *t* and *t* + 1, respectively.

### 2.2. Climate and Environmental Factors 

Several climate and environmental factors were used as explanatory factors in LSTM modeling, including daily land surface temperature (dLST), night land surface temperature (nLST), normalized difference vegetation index (NDVI), enhanced vegetation index (EVI), total rainfall (R), temperature (T), and relative humidity (RH) (Table 1), which have been used for predicting dengue risk in previous studies [4,30]. In order to generate the weekly composite for each factor during 2007–2019, we first selected the data covering our study area, with daily or sub-daily temporal resolution in the GEE platform. Two LST factors (dLST_mean_ and nLST_mean_) were derived from the MODIS MOD11A1 product with daily temporal resolution and 1000 m spatial resolution [31]; two vegetation indices (NDVI_mean_ and EVI_mean_) were derived from the MODIS MOD09GA product with daily temporal resolution and 500 m spatial resolution [32]; total rainfall (R_sum_) was derived from the Tropical Rainfall Measuring Mission (TRMM) 3B42 product with 3-hourly temporal resolution and 0.25 × 0.25 degree spatial resolution [33]; both mean temperature (T_mean_) and mean relative humidity (RH_mean_) were derived from the Global Land Data Assimilation System Version 2.1 (namely GLDAS-2.1), which is a global, ready-to-use dataset of land surface states and fluxes with daily temporal resolution and 0.25 × 0.25 degree spatial resolution and generated using satellite- and ground-based observational data, land surface modeling and data assimilation techniques [34]. We then created a suite of weekly composites according to the start date and end date of epi weeks and each weekly composite gives the value per pixel.

Considering both human-dominated areas during 2007–2019 and flight range of dengue vectors (i.e., *Aedes aegypti* and *Aedes albopictus*) reported in previous studies [35,36], we used the impervious map of 2013 and defined a buffer of 1 km around urban land pixels to delineate the area of dengue transmission in the FDB. We obtained a time series for each factor by spatially aggregating the pixel values of the weekly composite covering buffer zone according to the algorithms listed in Table 1. We tested the variance of individual factors and the correlation between two factors to filter the climate and environmental factors, and the factors having low variance (i.e., less than 0.02) and high correlation with others (i.e., greater than 0.6 with *p*-value < 0.05) were not used in LSTM modeling.

### 2.3. LSTM

The core idea of the common LSTM is to add the concept of a forgetting gate to the ordinary Recurrent Neural Network (RNN) unit to save historical information in order to achieve better training results. In an ordinary RNN, the hidden layer at each moment is determined not only by the input layer at that moment, but also by the hidden layer at the previous moment, and generally the neural unit weight matrix at each moment is the same. When the input is too large, ordinary RNN will have the problem of gradient disappearance and explosion due to too much memory. The idea of the forgetting gate of LSTM was created to solve this problem, because it has the function of selective storage. In LSTM, there are three types of gates: forget gates, input gates and output gates. LSTM can be regarded as an evolution of ordinary RNN units. The ordinary RNN unit has only the unit h that can be regarded as a short-term memory, while the LSTM adds a memory unit C that stores past information. The forget gate is used to process the information in the previous state. Its formula is expressed as:

(2) $$f_{t}=\sigma \left(W_{f}\cdot \left[h_{t-1},x_{t}\right]+b_{f}\right)$$

where σ represents the sigmoid function, $W_{f}$ is the weight matrix of the unit, $h_{t-1}$ represents the history information of the previous unit and $b_{f}$ represents the offset matrix of the unit. 

The input gate updates the state of the memory cell through a weighted sum operation of the input and the memory cell. The formula is often expressed as:

(3) $$i_{t}=\sigma \left(W_{i}\cdot \left[h_{t-1},x_{t}\right]\;+\;b_{i}\right)$$

(4) $$\tilde{C_{t}}=tanh\left(W_{C}\cdot \left[h_{t-1},x_{t}\right]\;+\;b_{C}\right)$$

(5) $$C_{t}=f_{t}*C_{t-1}+i_{t}*\tilde{C_{t}}$$

where $W_{i}$ and $b_{i}$ are the weight matrix and paranoia matrix of the unit, respectively. $C_{t}$ represents the state of the memory unit. 

After obtaining the new memory cell state through the above update formula, the final output gate determines the new output and updates the historical state. The formula is expressed as:

(6) $$o_{t}=\sigma \left(W_{o}\cdot \left[h_{t-1},x_{t}\right]+b_{o}\right)$$

(7) $$h_{t}=o_{t}*\tan h\left(C_{t}\right)$$

Since LSTM is used for classification problems, a single neuron is added to the last layer to obtain the predicted label, and the loss function uses the error between the real output $y_{t}$ and the predicted label.

### 2.4. Multi-Step-Ahead LSTM Modeling, Training, Validation and Testing Sets

The time series of historical dengue data, climate and environmental factors, and time-differencing natural log-transformed weekly dengue cases (i.e., the dependent factor used in this study), were combined to generate a dataset, which was divided into training, validation, and testing set, with the data for 2007–2015 as the training set, data for 2016–2017 as the validation set, and data for 2018–2019 and peak season in 2019 (i.e., January to August in 2019) as the testing set. The validation set was used to fix the parameters of LSTM (i.e., number of units, epoch, batch size, learning rate, and dropout rate) and the testing set was used to evaluate the generalization of LSTM. Moreover, in order to examine the role of historical dengue data and GEE-based external factors in dengue prediction, we defined multi-step-ahead forecast scenarios (i.e., 1- to 12-week-ahead) with two groups of input features (i.e., LSTM with climate and environmental factors and LSTM with historical dengue data and climate and environmental factors). A total of 24 LSTM models of dengue risk prediction were generated. Finally, the predicted value in target week and the number of weekly dengue cases in previous weeks were used to compute the number of weekly dengue cases in target weeks.

### 2.5. Model Evaluation 

In order to select the best LSTM model of dengue risk prediction, we first quantified the model accuracy based on the predicted and actual time-differencing natural log-transformed weekly dengue cases in testing set by computing the root mean squared error (RMSE) and mean absolute error (MAE) as follows [37]:

(8) $$\mathrm{RMSE}=\sqrt{\frac{1}{n}\overset{n}{\underset{i=1}{\sum }}{\left(o_{i}-y_{i}\right)}^{2}\;}$$

(9) $$\mathrm{MAE}=\frac{1}{n}\sum_{i=1}^{n}\left|o_{i}-y_{i}\right|$$

where $o_{i}$ represents the observed value for epi week *i*, and $y_{i}$ represents the predicted value for epi week *i*. In these models, the larger the indices value, the larger the error and the worse the model effect.

Then, we applied the Dropout method to examine the uncertainty of 1- to 12-week ahead LSTM models, which has been used in estimating the uncertainty of LSTM-based disease risk prediction [38]. Specifically, based on the fixed parameters of LSTM and the testing set, we outputted 50 predictions by dropping a fixed percent of units randomly and computed the maximum, minimum, and mean of 50 predicted values for each epi week to generate the predicted interval. We analyzed whether the observed value fell within the predicted interval to examine the uncertainty of the LSTM model.

Moreover, we used ARIMA as a baseline to provide a point of comparison for understanding the performance of 1- to 12-week ahead LSTM models. ARIMA, a univariate time series prediction model, makes predictions based on the autoregression (namely *p*), non-seasonal difference (namely *d*), and moving average (namely *q*) of stationary historical data, and has been used as a baseline model in dengue risk prediction [21]. In this study, based on natural log-transformed weekly dengue cases, the *d* in ARIMA was determined using ADF and KPSS. The best *p* and *q* values were determined by computing the Autocorrelation Function (ACF) and Partial Autocorrelation Function (PACF). We quantified the accuracy of ARIMA models by computing RMSE and MAE in both 2018–2019 and peak period for dengue in 2019.

## 3. Results

### 3.1. Time Series of Historical Dengue Data and Input Climate and Environmental Factors

Figure 2b shows the temporal pattern of weekly numbers of reported dengue cases in the FDB during 2007–2019. Large outbreaks could be found in 2010, 2013, 2014, 2015, 2016, and 2019. There was a sharp increase in weekly dengue cases in 2019. For each year, the epidemic season was mainly from February to May. Table 2 presents the results of the ADF test and KPSS test, and only the time-differencing natural log-transformed weekly dengue cases are stationary for both tests. 

**Figure 3:**
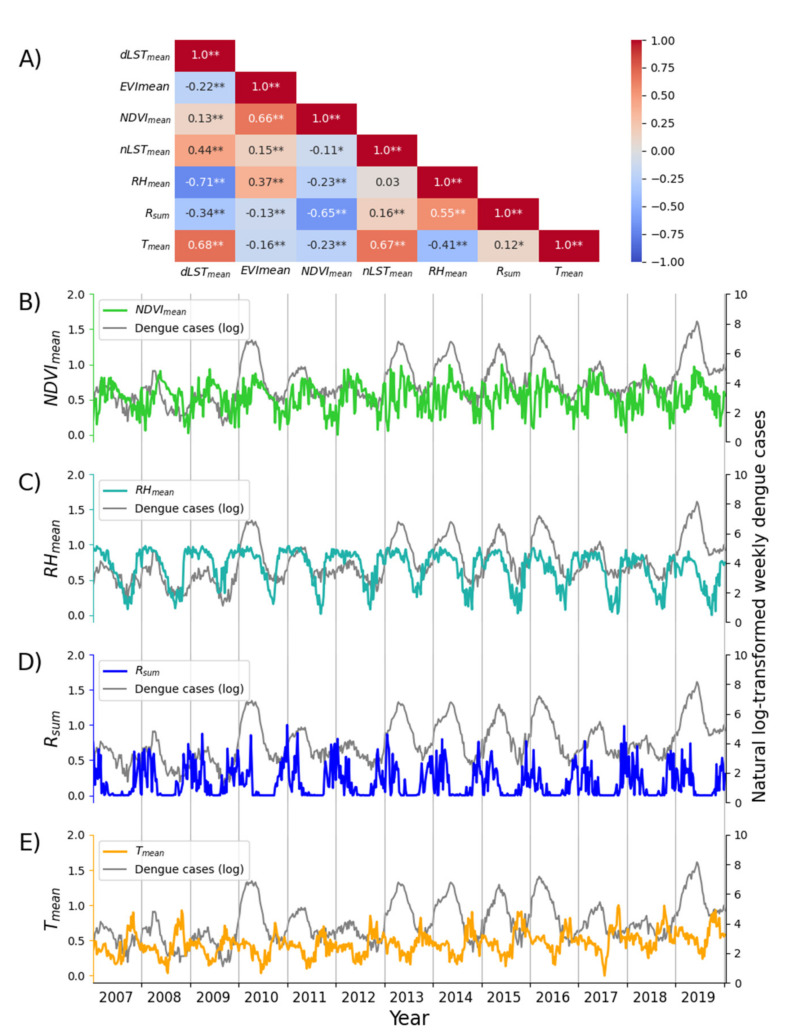
The correlations among the climate and environmental factors (**A**) and the temporal pattern of time series of the natural log-transformed weekly dengue cases and four selected driving factors (NDVI_mean_, RH_mean_, R_sum_, and T_mean_) during 2007–2019 (**B**–**E**). One asterisk (*) and two asterisks (**) in (**A**) represent a *p*-value of correlation coefficient less than 0.05 and 0.01, respectively.

Moreover, the correlations among the individual climate and environmental factors are presented in Figure 3a. The variance of all these factors is greater than 0.02. Based on these estimates, NDVI_mean_, RH_mean_, R_sum_, and T_mean_ were included in LSTM modeling. Figure 3b–3e presents the temporal patterns of the natural log-transformed dengue cases per epi week and the four selected factors during 2007–2019. 

### 3.2. Outcomes of LSTM Modeling

The LSTM networks used in this study were modeled using TensorFlow (version 2.0.0) and all the LSTM models used the same set of parameters (Table 3). All experiments were implemented in Python 3.6.5 that were run in 64-bit Windows with a 3.6 GHz, Intel Core i7-9700K CPU. Table 3 presents the parameters used for LSTM modeling with/without historical dengue information.

**Figure 4:**
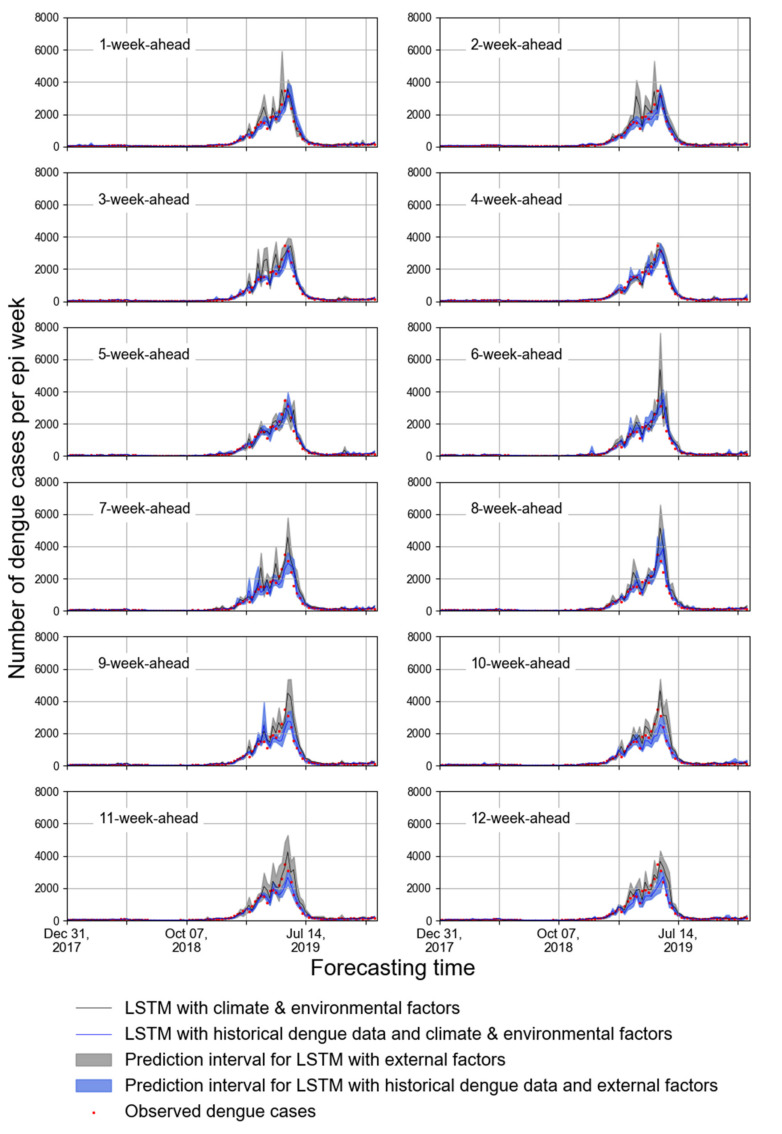
The 1- to 12-week-ahead prediction with two types of input features for the FDB. The red points represent the number of observed cases per epi week during 2018-2019. The blue interval represents the number of predicted cases per epi weekusing LSTM with historical dengue data, climate factors and environmental factors. The grey interval represents the number of predicted cases per epi week using LSTM with climate and environmental factors.

The predicted accuracies for forecasting the weekly dengue cases in the FDB during 2018–2019 and peak period in 2019 are presented in Table 4. Evidently, the ARIMA model had less accurate predictions in both periods. It is observed that the 4-week-ahead forecast using NDVI_mean_, RH_mean_, R_sum_, and T_mean_ obtained the lower values of RMSE and MAE; however, most of the predicted curves differed greatly from that of observed dengue cases (Figure 4). By contrast, while the historical dengue data were used as input features, 1-, 2- and 5-ahead forecasts obtained the lower values of RMSE and MAE, and the corresponding curves are similar to that of observed dengue cases. Moreover, Figure 4 shows that using historical dengue data as one of the input features could make the predicted curve fluctuation more stable.

## 4. Discussion

This study developed a framework for forecasting dengue cases per epi week based on the analyses of geospatial big data in the GEE platform and historical information-aided LSTM modeling. This framework permits the effective definition of the main area of dengue transmission according to remote sensing-based human-dominated areas, generating the time series of dengue risk predictors, directly forecasting the time-differenced natural log-transformed weekly dengue case and then computing the predicted number of dengue cases per epi week. 

Previous studies reported that climate data collected from weather stations are not practical to generate the time series of climate factors due to scare data, limited numbers, and uneven spatial distribution of stations in the target study area. These factors limit the choice of optimal spatial and temporal scales of risk prediction, accuracy, and the definition of prevention and control strategies [39,40]. By contrast, the GEE platform integrates an amount of geospatial data, diverse algorithms, and high-speed computing power, which provides greater convenience and more possibilities in the collection, preprocessing and spatio-temporal aggregation of multi-source data. It offers the opportunity to adjust the spatial and temporal scales according to different target study units (e.g., urban village, health unit, neighborhood, and city) and temporal resolutions of epidemiological data (e.g., daily, weekly, and monthly), respectively. The GEE’s climate and environmental data with daily or sub-daily temporal resolution satisfy the weekly dengue case count forecast. Compared with weekly dengue risk forecasting, there are more geospatial data options with the monthly or sub-monthly temporal resolutions to achieve monthly risk forecasting worldwide. Moreover, the annual data of global artificial impervious areas during 1985–2018 with 30 m spatial resolution and global human settlement layers for 1975, 1990, 2000 and 2016 have been generated in the GEE platform [41,42], which permits the definition of the main area of dengue transmission in a specific area worldwide and provides an opportunity to reflect the dynamic changes of dengue transmission areas.

Previous studies indicated the importance of using historical dengue data in dengue risk forecasting [43,44,45]. To improve the forecasting accuracy, many studies implemented the natural log-transformation for dengue time series to make it stationary. It should be noted that, by using only historical data in LSTM modeling, it is difficult to truly predict the information itself, as the autocorrelation in time series makes the LSTM underfitting (i.e., using the value at the previous time as the predicted one at the current time). Adding more external factors and using time-differenced dengue time series as target factors are two common ways to avoid underfitting. In this study, we used the time-differenced natural log-transformed weekly dengue cases as the target factor.

LSTM time series forecasting is suitable for predicting weekly dengue cases. It can capture the non-linearity and long-term dependency in the complex system of dengue transmission [5,21]. The parameter timestep in LSTM (i.e., the length of input time series) allow us to describe the impact of past climate and environmental conditions on mosquito populations. Then, LSTM is easy to integrate with different prediction scenarios (e.g., n-week-ahead prediction in this study), which might reflect the incubation period of dengue fever and the delay of dengue case notification. Moreover, the comparison between ARIMA and LSTM models also indicates the capacity of LSTM in the prediction of weekly dengue cases (Table 4).

Despite the involvement of historical data and external factors leading to higher accuracy (Table 4), we still cannot quantify the contribution of each feature in LSTM modeling. Future studies could focus on analyzing the importance of predictors (e.g., adding the self-attention in LSTM modeling [46]) in dengue risk prediction to understand the role of historical dengue data. Moreover, other RNN models, such as BiLSTM, GRU and Transformer, could be compared with LSTM and can be combined to generate the optimal prediction of dengue cases [19]. We could also integrate the prior knowledge of the response of mosquitos to climate and environmental conditions into the preprocessing of input time series to improve the model’s performance with climate and environmental factors [30]. Moreover, the geospatial big data analyses in dengue risk prediction only considered the climate and environmental factors. However, dengue transmission is affected by a complex interplay of human, climate, mosquito, and virus. Data related to immune population status [47], population movement [48], mosquito population [49,50], and cycle of dengue serotypes (DENV 1–4) [47] should be further collected and used in the deep learning model to improve the prediction accuracy.

It should be noted that this study focused on the prediction of the time series of weekly dengue cases using GEE-based external factors and historical dengue information and the prediction accuracy was evaluated using two common indices (i.e., RMSE and MAE). However, there are other needs for practical applications, such as predicting peak intensity and peak timing [21,51,52]. It thus needs to define the evaluation indices according to the different prediction targets.

There are some limitations for applying the proposed model in real applications. For example, many factors cause the misdetection of dengue cases in the FDB, such as people’s health seeking behavior, local health services’ misunderstanding of the importance of notifying dengue cases, and the lack of human resources for digitizing the notification forms [27]. Using reported cases could underestimate the real situation of dengue infection as asymptomatic and mild cases were most likely missed [53]. In addition, there is a time delay for the notified cases to be registered in the FDB due to the lack of human and technological resources and better integration in private healthcare [27]. These facts might impede the application of proposed models in this region using the proposed model as we directly predicted the change in dengue cases between two adjacent weeks and it needs to compute the number of dengue cases in target week based on the value of last week. Thus, optimizing the dengue surveillance system, improving the efficiency for dengue case notification, and raising awareness of seeking health care for dengue fever could greatly facilitate the application of the proposed model.

## 5. Conclusions

The accurate and timely dengue risk forecast enables enhancement of the effectiveness of dengue control. Multi-source data and interdisciplinary knowledge (e.g., epidemiology, remote sensing and geoinformation science) are needed to generate predictors of dengue risk at certain spatial and temporal scales that often impede the timely and accurate forecast of dengue risk. This study used GEE to rationally and efficiently generate the time series of dengue predictors according to the spatial pattern of urban land and the flight of *Aedes aegypti* and *Aedes albopictus.* It demonstrates the great potential of the GEE platform in epidemic prediction through the exploration of climate and environmental predictors based on geospatial big data. Then, using the change in dengue cases per epi week as outcomes, a framework of time-differenced dengue risk forecasting is proposed based on LSTM modeling with historical dengue cases, total rainfall, mean temperature, and mean relative humidity. Our findings show that the proposed framework can forecast dengue cases in the future successfully. This study efficiently and rationally explores the potential of geospatial big data and deep learning for advancing the infectious disease forecast.

## Figures and Tables

**Table 1:** Summary of explanatory factors and data sources used in this study.

Explanatory Factors	Unit	Algorithm	Data Sources and Spatio-Temporal Resolutions	
Log-transformed weekly dengue cases	Number	Sum	SINAN	weekly (epi week), city
dLST_mean_	°C	Average	MOD11A1	daily, 1000 m
nLST_mean_	°C	Average		
NDVI_mean_	-	Average	MOD09GA	daily, 500 m
EVI_mean_	-	Average		
R_sum_	mm	Sum	TRMM 3B42	3-hourly, 0.25 × 0.25 degree
T_mean_	°C	Average	GLDAS-2.1	daily, 0.25 × 0.25 degree
RH_mean_	%	Average	GLDAS-2.1	

**Table 2:** Results of the stationarity ADF test and KPSS test of time series of dengue data and external factors.

Dengue Data	ADF	KPSS
Weekly dengue cases	−6.28 *	0.399 **
Natural log-transformed weekly dengue cases	−5919 *	0.789 **
Time-differencing natural log-transformed weekly dengue cases	−5.67 *	0.068 *
NDVI_mean_	−7.875 *	0.061 *
RH_mean_	−7.662 *	0.293 *
R_sum_	−8.387 *	0.052 *
T_mean_	−7.497 *	1.008 **
1% level	−3.4401	0.739
5% level	−2.8658	0.463
10% level	−2.569	0.347

* Stationary ** Non-stationary.

**Table 3:** The parameters in LSTM models used in this study. Time step refers to the length of input features used to make predictions. Loss function measures the difference between predicted and observed values. Number of units refers to the number of units in the LSTM layer. Epoch represents the number of completed training using all data in a training set. Batch size refers to the size of the input data used to update LSTM parameters one time. Learning rate refers to the rate for updating LSTM parameters. Optimizer refers to the algorithm for updating parameters. Dropout rate is the percent of units in the LSTM layer that is randomly discarded in the model training. The two groups of LSTM parameters were fixed separately by comparing the RMSE and MAE computed, based on validation, dataset.

Parameters	LSTM with NDVImean, RHmean, Rsum and Tmean	LSTM with Historical Dengue Data, NDVImean, RHmean, Rsum and Tmean
Time step	12	12
Loss function	MSE	MSE
Number of units	64	64
Epoch	1150	2000
Batch size	12	12
Learning rate	0.005	0.001
Optimizer	Adam	Adam
Dropout rate	0.8	0.65

**Table 4:** Accuracy comparison of multi-step-ahead LSTM modeling with two groups of input features and ARIMA using root-mean-square error (RMSE) and mean absolute error (MAE). The two indices were computed based on the actual and predicted weekly changes in natural log-transformed dengue cases.

Model			2018–2019		2019 Peak Period	
			RMSE	MAE	RMSE	MAE
LSTM modeling	LSTM with *NDVI_mean_*, *RH_mean_*, *R_sum_*, and *T_mean_*	1-week	0.36	0.29	0.28	0.23
		2-week	0.35	0.28	0.30	0.23
		3-week	0.36	0.28	0.34	0.26
		4-week	0.32	0.25	0.22	0.18
		5-week	0.36	0.29	0.29	0.24
		6-week	0.36	0.29	0.31	0.25
		7-week	0.38	0.3	0.35	0.29
		8-week	0.37	0.29	0.36	0.28
		9-week	0.38	0.3	0.34	0.29
		10-week	0.36	0.29	0.34	0.27
		11-week	0.36	0.29	0.34	0.29
		12-week	0.36	0.27	0.31	0.25
	LSTM with historical dengue data, *NDVI_mean_*, *RH_mean_*, *R_sum_*, and *T_mean_*	1-week	0.35	0.27	0.23	0.20
		2-week	0.34	0.27	0.22	0.19
		3-week	0.34	0.27	0.25	0.20
		4-week	0.35	0.26	0.25	0.21
		5-week	0.34	0.27	0.22	0.19
		6-week	0.40	0.31	0.26	0.21
		7-week	0.37	0.30	0.28	0.22
		8-week	0.38	0.29	0.29	0.23
		9-week	0.38	0.29	0.32	0.27
		10-week	0.39	0.31	0.28	0.22
		11-week	0.34	0.27	0.28	0.23
		12-week	0.40	0.33	0.33	0.28
Baseline	ARIMA (3, 1, 2)		1.60	1.18	2.68	2.51
